# Functional and Cosmetic Outcome after Reconstruction of Isolated, Unilateral Orbital Floor Fractures (Blow-Out Fractures) with and without the Support of 3D-Printed Orbital Anatomical Models

**DOI:** 10.3390/jcm10163509

**Published:** 2021-08-09

**Authors:** Guido R. Sigron, Marina Barba, Frédérique Chammartin, Bilal Msallem, Britt-Isabelle Berg, Florian M. Thieringer

**Affiliations:** 1 Department of Oral and Cranio-Maxillofacial Surgery, University Hospital Basel, CH-4031 Basel, Switzerland; marina.barba@unibas.ch (M.B.); bilal.msallem@usb.ch (B.M.); isabelle.berg@usb.ch (B.-I.B.); f.thieringer@unibas.ch (F.M.T.); 2 Medical Additive Manufacturing Research Group (Swiss MAM), Department of Biomedical Engineering, University of Basel, CH-4123 Allschwil, Switzerland; 3 Department of Clinical Research, Basel Institute for Clinical Epidemiology and Biostatistics, University Hospital Basel, University of Basel, CH-4031 Basel, Switzerland; frederique.chammartin@usb.ch

**Keywords:** orbital fracture, blow-out fracture, orbital reconstruction, functional outcome, patient-specific implant, printed anatomical model

## Abstract

The present study aimed to analyze if a preformed “hybrid” patient-specific orbital mesh provides a more accurate reconstruction of the orbital floor and a better functional outcome than a standardized, intraoperatively adapted titanium implant. Thirty patients who had undergone surgical reconstruction for isolated, unilateral orbital floor fractures between May 2016 and November 2018 were included in this study. Of these patients, 13 were treated conventionally by intraoperative adjustment of a standardized titanium mesh based on assessing the fracture’s shape and extent. For the other 17 patients, an individual three-dimensional (3D) anatomical model of the orbit was fabricated with an in-house 3D-printer. This model was used as a template to create a so-called “hybrid” patient-specific titanium implant by preforming the titanium mesh before surgery. The functional and cosmetic outcome in terms of diplopia, enophthalmos, ocular motility, and sensory disturbance trended better when “hybrid” patient-specific titanium meshes were used but with statistically non-significant differences. The 3D-printed anatomical models mirroring the unaffected orbit did not delay the surgery’s timepoint. Nonetheless, it significantly reduced the surgery duration compared to the traditional method (58.9 (SD: 20.1) min versus 94.8 (SD: 33.0) min, *p*-value = 0.003). This study shows that using 3D-printed anatomical models as a supporting tool allows precise and less time-consuming orbital reconstructions with clinical benefits.

## 1. Introduction

Fractures of the orbit are common injuries of the midface [1]. They can cause changes in the orbital structures and entrapments of vital tissues resulting in the most common symptoms diplopia, impairment of ocular motility, and enophthalmos [2]. The treatment’s primary goal is to restore the orbit’s original volume and contour to achieve normal eye function and aesthetics [3].

Various indications, available methods, and materials for reconstructing the orbital floor defects are still controversially discussed nowadays. To date, for example, there are no uniform recommendations for surgical intervention of orbital floor fractures. The indication for surgery depends on many factors and must be decided individually for each patient after careful evaluation of clinical and radiographic findings [4,5,6]. Furthermore, there is no explicit agreement about the ideal material for orbital reconstructions. The material should be similar to the orbital bone, easy to handle, and provide a lifelong stable result [7]. Titanium shows chemical similarity to the calcium in the bone structure and has convinced it as a successful implant material with its high availability and advantageous properties such as high biocompatibility, low thickness, and great malleability and stability [7,8,9]. Therefore it is considered a superior material for orbital reconstruction [8]. Reconstruction with titanium is still often performed conventionally, requiring the titanium meshes to be bent and adjusted during surgery according to the size and shape of the fractured orbital floor [10]. Due to the complex three-dimensional anatomy of the orbit and the limited surgical access that restricts vision, orbital reconstructions remain a significant challenge [11,12].

Modern technologies like medical additive manufacturing (MAM), also called medical three-dimensional (3D) printing, allow the fabrication of individual three-dimensional (3D) anatomical orbital models to better understand the specific anatomy and the pre-operative planning of the surgical intervention. With the introduction of in-house 3D-printers, orbital models’ production has become more cost-effective over the years and is gaining more popularity for clinical use [13,14,15]. The titanium meshes that are preformed according to the patient’s orbital model are an extremely precise, less time-consuming, and less tissue-damaging technique for orbital restorations. The accuracy of the repair with preformed titanium mesh is shown to be in a range within 1 mm compared to the virtually planned reconstruction by mirroring the unaffected, contralateral orbit [16,17]. Another possibility of individualizing implants for orbital reconstruction is computer-aided design and computer-aided manufacturing (CAD/CAM), also showing accurate restoration of the orbital volume and shape [18,19]. Therefore, 3D-printing is a promising, cost-efficient supporting tool that can improve the outcome of complex and demanding orbital reconstructions.

This retrospective cohort study aimed to compare the functional outcome after reconstruction of isolated, unilateral orbital floor fractures with intraoperative bent titanium meshes and the use of preformed patient-specific titanium meshes based on an in-house 3D-printed anatomical model. Furthermore, the assessment focused on surgery time and pre- and post-operative hospital stay.

## 2. Experimental Section

**Figure 1:**
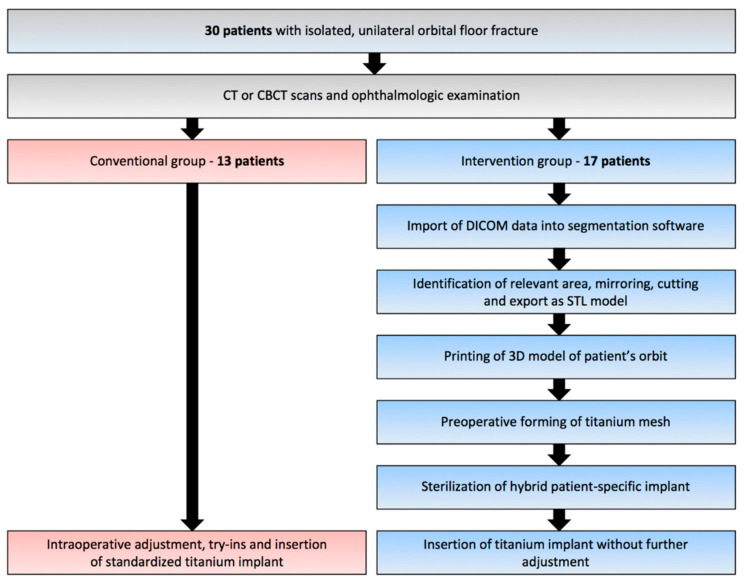
Study design and treatment protocol.

The retrospective study was performed at the Department of Oral and Cranio-Maxillofacial Surgery, University Hospital Basel in Switzerland, following approval of the Ethics Committee of Northwest and Central Switzerland (EKNZ; Project-ID: 2019-00260). The study design and treatment protocol are shown in Figure 1. The inclusion criteria were: (a) isolated, unilateral orbital floor fracture; (b) complete medical records inclusive pre- and post-operative ophthalmologic examination as well as computed tomography (CT) or cone-beam computed tomography (CBCT) scans; (c) surgical reconstruction with orbital floor mesh. Supported by literature, indications for surgical reconstruction were as follows: fractures with defects ≥ 3 cm^2^, enophthalmos ≥ 2 mm, dislocated fractures, herniation of soft tissue, and/or muscle entrapment [5,6]. From May 2016 to November 2018, 32 patients underwent reconstruction of isolated, unilateral orbital floor fracture with a titanium mesh plate, of whom 30 patients (15 women, 15 men) satisfied the inclusion criteria of the present study.

In our previous study twenty-two of the thirty patients were assessed with a particular focus on the pre- and post-operative orbital volume, the fracture area, the maximum fracture collapse, the surgery time, and the comparison of the efficacy of intraoperative bending of titanium mesh with the efficacy of pre-contoured “hybrid” patient-specific titanium mesh [20]. The other eight patients could not be included in the previous study due to non-evaluable radiological data.

In the conventional group, 13 patients received standardized titanium mesh plates (MatrixMIDFACE, DePuy Synthes, Solothurn, Switzerland or MODUS OPS 1.5, Medartis, Basel, Switzerland) using the conventional method of intraoperative manual bending and adjusting to fit the extent and shape of the fracture.

In the intervention group, 17 patients were reconstructed with titanium mesh plates (MatrixMIDFACE, DePuy Synthes, Solothurn, Switzerland or MODUS, Medartis, Basel, Switzerland), preformed with the support of a patient-specific 3D model of the region of interest (ROI). Patients were randomly assigned to one of the two groups.

The medical records were reviewed retrospectively for the following clinical and surgical parameters: sex, age, cause of injury, pre- and post-operative volume-related symptoms, use of 3D-printed anatomical models for pre-operative adjusting of the plates, duration of surgery, the time interval between trauma and surgery, admission and surgery and between surgery and discharge, and course of follow-up.

### 2.1. Virtual Planning and Manufacturing of the 3D Model

**Figure 2:**
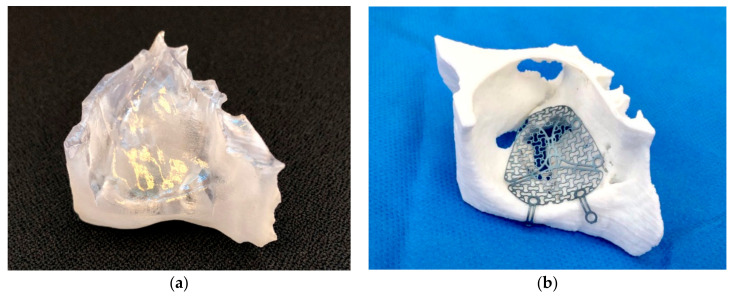
Overview of the used materials and techniques: (**a**) individual, virtually reconstructed right patient orbit (region of interest) printed with Med610 material; (**b**) individualized titanium mesh (Medartis MODUS OPS 1.5) on a 3D-printed right orbital model for the intervention group.

For the virtual design of the patient’s anatomical orbital model, the pre-operative computed tomography (Siemens Somatom, Siemens Healthcare GmbH, Erlangen, Germany) or cone-beam computed tomography (Carestream CS 9300, Carestream Health, Inc., Rochester, NY, USA) images were imported in digital imaging and communications in medicine (DICOM) format into the visualization and segmentation software Mimics Innovation Suite v. 20–21 (Materialise, Leuven, Belgium). In the patient’s non-fractured orbit, the anatomical area to be reconstructed was defined, mirrored, cut to the appropriate size, and exported as a file in standard tessellation language (STL) format. With the slicing software of the corresponding desktop printers MakerBot Replicator+ (MakerBot Industries, Brooklyn, New York, NY, USA) or Stratasys Objet30 Prime (Stratasys, Ltd., Eden Prairie, MN, USA), a 3D anatomical model of each patient’s orbit was printed from polylactic acid (PLA) filaments (MakerBot) or Med610 biocompatible resin (Stratasys). Before reconstruction surgery, a standardized orbital plate (MatrixMIDFACE, DePuy Synthes, Solothurn, Switzerland or MODUS OPS 1.5, Medartis, Basel, Switzerland) was manually shaped and adjusted to fit the extent of the defect based on the fabricated patient-specific 3D orbital template and sterilized (Figure 2b).

### 2.2. Surgical Procedure

The orbital reconstructions were performed under general anesthesia by experienced oral and maxillofacial surgeons of the Department of Oral and Cranio-Maxillofacial Surgery, University Hospital Basel in Switzerland. After accessing the fracture through a mid-eyelid (n = 28) or transconjunctival incision (n = 1) and in one case through a laceration, the herniated orbital tissue was reduced. Either freehand bent (conventional group) or pre-bent (intervention group) titanium mesh plates were inserted to cover the fracture zone and fixated with titanium screws to the inferior orbital rim. The patient-specific titanium plates did not need any further manual adjustment. Before skin closure, surgeons performed a forced duction test to verify the passive eye movement to every site. The surgery time was recorded, defined from the moment of incision to suture.

### 2.3. Ophthalmic Examination

All 30 patients received an ophthalmic examination before surgery and post-operatively by an ophthalmologist. Position of the globe (normal, enophthalmos, exophthalmos), diplopia (none, in upgaze, in other directions), limitations of eye movement (none, in upgaze, in other directions), radiologically herniation of fat tissue (yes, no), and sensory disturbances of the infraorbital nerve (yes, no) were noted. Enophthalmos and exophthalmos were defined as posterior and anterior displacement of the globe, respectively, with a difference of two or more millimeters (≥2 mm) compared to the unaffected eye. Both were measured and confirmed with Hertel exophthalmometry. Limitations of the eye globe motility were examined with the H pattern test.

Complications were considered as functional limitations in gazes within the normal field of vision without the tendency of regression and with the need for further intervention. Post-operative symptoms that were mild or occurred only in extreme peripheral gaze and the patient did not even notice that these symptoms, which subjectively were not considered complications.

### 2.4. Statistical Analysis

The statistical analysis was carried out using the R software version 3.6.1 (R Foundation for Statistical Computing, Vienna, Austria). Results are presented as mean, standard deviation (SD), or range, or as percentage. The comparison between the two groups was evaluated with Fisher’s exact test for categorical variables and Student’s *t*-test for continuous variables. A *p*-value of less than 0.05 was considered statistically significant.

## 3. Results

Of the 32 patients undergoing orbital reconstruction of isolated, unilateral orbital floor fracture with a titanium mesh, 30 (15 female and 15 male patients, mean age 51.2 (SD 20.4) years, range from 20 to 91 years) met the inclusion criteria. The descriptive statistics of the patients are shown in Table 1.

### 3.1. Surgery Time and Hospital Stay

**Figure 3:**
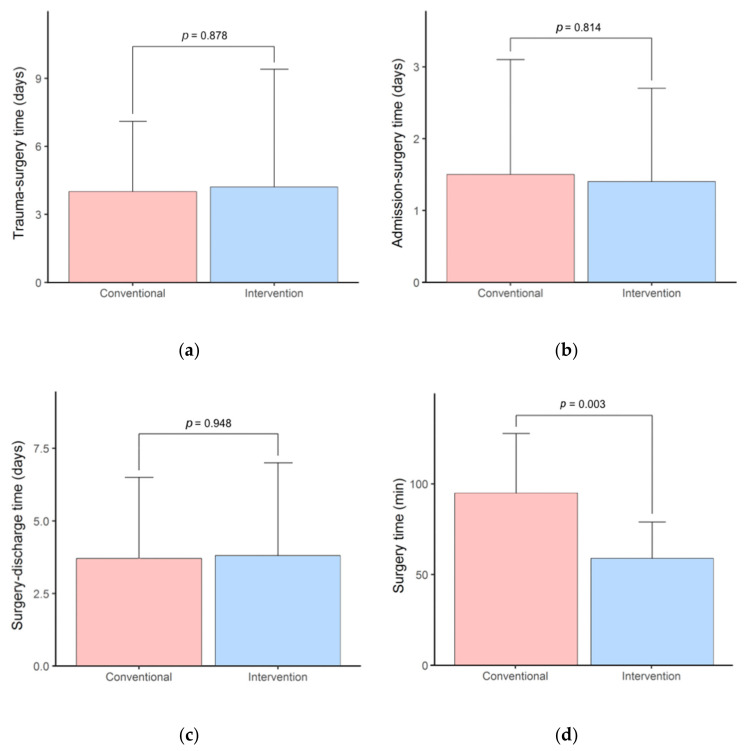
Comparison of different times between the conventional and intervention group: (**a**) time from trauma to surgery (days); (**b**) time from admission to surgery (days); (**c**) time from surgery to discharge (days); (**d**) duration of surgery (minutes) from incision to suture.

The mean (SD) time from trauma to surgery was in the conventional group 4.0 (3.1) days and in the intervention group 4.2 (5.2) days. The time from admission to surgery was 1.5 (1.6) days and 1.4 (1.3) days, respectively. The time from surgery to discharge was 3.7 (2.8) days in the conventional and 3.8 (3.2) days in the intervention group. The mean (SD) duration of the surgery was significantly reduced by 35.9 min in the intervention group (58.9 (SD: 20.1) min) compared to the conventional group (94.8 (SD: 33.0) min, *p*-value = 0.003) (Figure 3).

### 3.2. Ophthalmic Examination

The most common post-operative symptoms were diplopia, restriction in ocular motility, and sensory disturbance such as hypesthesia of the infraorbital nerve. An overview of the pre- and post-operative ophthalmologic findings is shown in Table 2.

Pre-operative enophthalmos was present in 6 patients (20.0%) and resolved entirely after surgery in both groups and did not develop after reconstruction in any case. The intervention group showed one case with new exophthalmos after surgery as a result of the post-operative swelling. 

Of the 19 patients who presented pre-operatively with diplopia (63.3%), 12 (40.0%) were in the intervention group and 7 (23.3%) were in the conventional group. The intervention group recorded complete resolution in 9 patients (75.0%) and remaining diplopia at extreme eye movements in 3 patients (25.0%). In comparison, the conventional group recorded full resolution of diplopia in 5 patients (71.4%), residual diplopia in extreme gaze in one patient (14.3%), and no resolution at all-in-one patient (14.3%). New diplopia in extreme gaze positions developed after surgery in two cases of the conventional group and one of the intervention groups. One patient of the conventional group did not show a spontaneous eye-opening and could not be evaluated regarding diplopia.

Of the 14 patients with pre-operative ocular motility impairment (46.7%), 9 (30.0%) were in the intervention group and 5 (16.7%) in the conventional group. The intervention group recorded 7 (77.8%) patients with a complete improvement of ocular motility and 2 (22.2%) patients with remaining mild ocular impairment compared to the conventional group, where 3 (60.0%) patients experienced a complete improvement of ocular motility, 1 (20%) patient a persistent but mild ocular motility impairment and also 1 (20.0%) patient no improvement.

In both groups, pre-operative sensory disturbance ultimately improved in 50.0% of the patients (2 cases in the conventional and 5 in the intervention group) and persisted in the other 50.0%.

Major complications such as retrobulbar hematoma did not occur at all. As an adverse event, ectropion was recorded in 2 cases from each group and regressed over time.

Overall, the examined symptom resolution and favorable clinical outcome trended better in the intervention group but did not achieve statistical significance, as shown in Table 3. Pre- and post-operative volume-related symptoms such as diplopia and eye movement limitations did not occur in the gaze’s primary position.

## 4. Discussion

The purpose of this study was to evaluate and compare the functional outcome in terms of diplopia, enophthalmos, ocular motility, and sensory disturbance after orbital reconstructions with standardized and a “hybrid” patient-specific implant, which was pre-bent on a 3D-printed orbital model. In a previous study, most of the patients have been assessed regarding pre- and post-operative orbital volume and surgery time, assuming that the “hybrid” patient-specific implants lead to more precise orbital reconstructions and a shortened surgery time [20].

Many guidelines for achieving the best surgical outcome after orbital floor reconstruction remain controversial to this day. Different materials have been introduced over time, starting from bone grafts to alloplastic materials such as titanium or porous polyethylene. The advantages and disadvantages of the most used materials for reconstruction are described in detail in the literature [7,8,9,21]. Initially, autogenous bone grafts were considered the best reconstruction material because of the bone’s similarity to be reconstructed and low susceptibility to infections and immune reactions. However, it also has disadvantages such as the risk of resorptions, especially for fragile grafts, donor site morbidity, and increased surgery time [7,21]. Titanium has proven to be a very successful implant material due to its high biocompatibility, low thickness, and great malleability and stability [7,8,9]. Other materials such as polydioxanone (PDS) foil are also commonly used but seem to have a higher risk of retrobulbar hematoma [22] and are only advised for use in more minor orbital floor defects.

Another essential factor in achieving the best functional and aesthetic results is the timing of surgical reconstruction. Although the recommendations are not uniformly regulated, a common opinion is early surgical repair within two weeks, depending on critical symptoms such as symptomatic diplopia, early enophthalmos, and herniation of orbital tissue [4,6]. After reconstruction, symptoms or complications may occur, for example, diplopia, restrictions in ocular motility, enophthalmos, or sensory disturbance, especially of the infraorbital nerve [23]. In accordance with other studies, an improvement of post-operative symptoms can be expected within 12 months of follow-up [24,25]. This time frame should be respected in any case before revision surgery is considered. Typically, all patients are followed up until symptoms have resolved. The present study’s follow-up times ranged from only one day to almost two years, with a mean follow-up rate of approximately six months. Patients who experienced post-operative symptoms in extreme positions of gaze described them as unnoticeable and, therefore, did not feel restricted in their daily lives, causing them not to come to follow-ups after a few months.

New technologies such as the pre-operative manufacturing of 3D-printed models are gaining more importance in the medical field. They are considered helpful in diagnostics, pre-operative treatment planning, or better visualization of the patient’s complex anatomy [13,26,27]. Sterilization processes are no limitation of use in daily clinics anymore [28]. With the support of an individually 3D-printed orbital model, the titanium plates can be performed to the exact shape of the orbital floor geometry, which allows a more accurate restoration of the premorbid contour [16,17,20]. Hence, pre-operative symptoms such as enophthalmos, diplopia, and ocular motility restrictions can be resolved entirely or at least be improved so that no further intervention is necessary [16,29,30]. Orbital reconstructions with individually preformed titanium meshes achieved similar or better results than the conventional reconstruction method [20,23,24,31,32]. The study of Holmes and Schlittler [33] showed a statistically significant improvement using patient-specific implants, especially in larger and more complex fractures. In our previously published study, which includes most of the present study’s patients, the pre- and post-operative orbital volume analysis showed a statistically significant absolute volume difference in the conventional group.

In contrast, the intervention group’s difference was not statistically significant, suggesting that “hybrid” patient-specific implants allow for more accurate orbital reconstructions [20]. The present study showed a slightly better functional outcome when a prebent titanium mesh is used compared to the conventional method. Although the comparison with the literature is limited due to different interpretations and definitions of the functional outcome, promising results and benefits can be shown in the use of patient-specific titanium meshes. The study of Kozakiewicz et al. [24] presented a better ophthalmic long-term outcome for individual orbital implants. Zimmerer et al. [23] reported no significant differences in post-operative diplopia and limited ocular motility between the two groups. Kim et al. [31] showed in their study a higher rate of post-operative resolution or persistence of only mild symptoms in the group with pre-bent patient-specific implants, whereas the conventional group also experienced moderate to severe post-operative symptoms. Analogous to this is the functional outcome in the present study. The 3D-group showed minimally better ophthalmological results and either complete resolution or clear improvement of the symptoms, with no statistically significant differences, which is in line with recent literature (Table 2 and Table 3). Unlike the report of Zieliński et al. [34], our results did not show any impact on the length of post-operative hospital stay when individualized titanium mesh plates were used. Our results showed that the difference between the two groups regarding all three measured and evaluated time intervals (trauma to surgery, admission to surgery, and surgery to discharge) was statistically not significant at the 5% level, suggesting that the use of patient-specific implants did not influence the pre- and post-operative hospital stay (Figure 3). Furthermore, with the additional support of surgical navigation, the placement and insertion of the orbital implants can be controlled, which might allow for an even more precise restoration of the orbital volume [23].

According to earlier studies, an important statistically significant difference between the two groups was the reduction of surgery time in the intervention group due to the precisely preformed titanium mesh on the anatomical orbital model with no need for further adjustment during surgery [20,23,32,34]. In this study, the time of implant insertion, defined by the point of incision to suture, was significantly shortened on average by 35.9 min and is similar to our previously published data due to partially the same patients [20]. Greater accuracy of the titanium implant prevents bad adaptation and minimizes the number of try-ins, leading to minor damage to the surrounding tissue [16,32,35]. Shorter operation time can decrease intraoperative complications by reducing the time of general anesthesia. Consequently, 3D-printing, as described in this study, can save surgery costs by reducing operating room time [36,37]. The cost savings depend on the hospital and country, respectively [20,38,39]. 

Commonly reported disadvantages of 3D-printed models were high costs and long production time [11,17]. There is also the need for external printing service and the resulting long delivery time [29]. These factors may lead to limited use of rapid prototyping in the medical area. Low-cost 3D-printers have recently been introduced, providing a cost- and time-effective workflow and a more widely available alternative for daily clinical use [13,14,15]. In the present study, the models were fabricated using an in-house 3D-printer with a cost-effective workflow. Virtual planning, 3D-printing, and pre-operative bending of the titanium plate take less than 2 h. Therefore, the reconstruction technique described did not delay the timing of surgery, which is less expensive and time-consuming than the external printing of standard stereolithographic models [35]. Nowadays, an entry-level, in-house 3D-printer can be purchased at a low cost than a professional printer [14,36]. The accuracy of low-cost, in-house 3D- printed models has proven to be satisfying for daily oral and maxillofacial surgery procedures, such as educational purposes, pre-operative surgery planning, and bending of plates [13,14,20,37]. 

The present study has several limitations. First, the small sample size limits the statistical power of our analysis and the validity of our promising findings regarding the benefit of the presented innovative reconstruction method in terms of ophthalmic outcome should be confirmed by more extensive studies. Second, the orbital reconstructions were performed by plural surgeons, which might increase the inter-operator variability and thereby affect the outcome. Third, the period of follow-ups was not uniform and varied very much. The availability of the patients for post-operative control appointments is not always guaranteed. Most of them will no longer appear for follow-up, as they feel subjectively free of symptoms. We have still included these patients to evaluate surgical parameters and descriptive patient data, even if this might affect the functional results. Finally, an in-depth evaluation of the cost savings through the reduced operating room time and a cost comparison at international level has not been feasible due to the difficulty of addressing the different standards. Therefore, the statement that 3D- printing is financially viable for everyone cannot be generalized.

## 5. Conclusions

Patient-specific 3D-printed orbital models can be a precious and economically viable tool for repairing orbital floor fractures and have recently become more generally available in the medical field. The mirror-imaged 3D-printed orbital models provide a better insight into the already complex anatomy of the orbit and serve as a template for pre-operative bending of orbital titanium plates, so-called “hybrid” patient-specific implants. This allows for greater contour accuracy and less invasive insertion without several fitting attempts or malpositioning of the implant. Compared to the classical treatment with intraoperative manual titanium mesh bending, the results of the present study show that “hybrid” patient-specific titanium implants significantly shorten the surgery time, do not delay the time of surgery thanks to in-house 3D-printing, and are slightly more effective in terms of ophthalmic outcome, even though not significantly. 

## Figures and Tables

**Table 1:** Descriptive statistics of the study sample.

Variable	Overall	Conventional Group	Intervention Group
Number of patients, n (%)	30 (100)	13 (43.3)	17 (56.7)
Age (years)			
Mean (SD)	51.2 (20.4)	49.6 (19.1)	52.4 (21.8)
Range	20–91	21–79	20–91
Sex, n (%)			
Female	15 (50)	4 (30.8)	11 (64.7)
Male	15 (50)	9 (69.2)	6 (35.3)
Cause of injury, n (%)			
Fall	16 (53.3)	7 (53.8)	9 (52.9)
Assault	9 (30.0)	5 (38.5)	4 (23.5)
Sports accident	3 (10.0)	1 (7.7)	2 (11.8)
Vehicle accident	1 (3.3)	0 (0.0)	1 (5.9)
Work accident	1 (3.3)	0 (0.0)	1 (5.9)
Site of injury, n (%)			
right	11 (36.7)	2 (15.4)	9 (52.9)
left	19 (63.3)	11 (84.6)	8 (47.1)
Follow-up (SD) (days)	196 (233)	191.2 (279)	200 (200)

**Table 2:** Results of the pre- and post-operative ophthalmic examinations in the two groups, n (%).

	Conventional Group				Intervention Group			
	Pre-Operative		Post-Operative		Pre-Operative		Post-Operative	
Position of globe								
Normal	10	(76.9)	12	(92.3)	12	(70.6)	16	(94.1)
Enopthalmos	2	(15.4)	0	(0.0)	4	(23.5)	0	(0.0)
Exophthalmos	1	(7.7)	1	(7.7)	1	(5.9)	1	(5.9)
Total	13	(100)	13	(100)	17	(100)	17	(100)
Diplopia								
None	5	(41.7)	8	(66.6)	5	(29.4)	13	(76.4)
Upgaze	3	(25.0)	2	(16.7)	3	(17.7)	2	(11.8)
Upgaze and other directions	4	(33.3)	2	(16.7)	9	(52.9)	2	(11.8)
Total	12	(100)	12	(100)	17	(100)	17	(100)
Motility impairment								
None	8	(61.5)	11	(84.6)	8	(47.0)	14	(82.3)
Elevation	3	(23.1)	1	(7.7)	6	(35.3)	1	(5.9)
Elevation and other directions	2	(15.4)	1	(7.7)	3	(17.7)	2	(11.8)
Total	13	(100)	13	(100)	17	(100)	17	(100)
Herniation of muscle								
None	10	(76.9)			11	(64.7)		
M. rectus inferior	3	(23.1)			6	(35.3)		
Total	13	(100)			17	(100)		
Herniation of fat tissue								
None	3	(23.1)			5	(29.4)		
Yes	10	(76.9)			12	(70.6)		
Total	13	(100)			17	(100)		
Sensory disturbances N. V2								
None	9	(69.2)	9	(69.2)	7	(41.2)	10	(58.8)
Yes	4	(30.8)	4	(30.8)	10	(58.8)	7	(41.2)
Total	13	(100)	13	(100)	17	(100)	17	(100)

N. V2: Maxillary nerve (second division of the trigeminal nerve).

**Table 3:** Favorable clinical outcome compared between the two groups.

Variable	Conventional Group	Intervention Group	*p*-Value *
Post-operative findings, n (%)			
No post-operative diplopia	8 (38.1)	13 (61.9)	0.335
No post-operative motility impairment	11 (44.0)	14 (56.0)	0.439
No post-operative sensory disturbance N. V2	9 (47.4)	10 (52.6)	0.473

* Statistically significant at *p* < 0.05. N. V2: Maxillary nerve (second division of the trigeminal nerve).

## Data Availability

The data presented in this study are available on request from the corresponding author.

## Abbreviations

CAD/DAM	Computer-aided design/computer-aided manufacturing
3D	Three-dimensional
ROI	Region of interest
DICOM	Digital Imaging and Communications in Medicine
MAM	Medical Additive Manufacturing
MIMICS	Materialise Interactive Medical Image Control System
STL	Standard Tessellation Language
PLA	Polylactic acid
SD	Standard deviation
PDS	Polydioxanone
